# Maternal mental health in primary care in five low- and middle-income countries: a situational analysis

**DOI:** 10.1186/s12913-016-1291-z

**Published:** 2016-02-16

**Authors:** Emily C. Baron, Charlotte Hanlon, Sumaya Mall, Simone Honikman, Erica Breuer, Tasneem Kathree, Nagendra P. Luitel, Juliet Nakku, Crick Lund, Girmay Medhin, Vikram Patel, Inge Petersen, Sanjay Shrivastava, Mark Tomlinson

**Affiliations:** Alan J Flisher Centre for Public Mental Health, Department of Psychiatry and Mental Health, University of Cape Town, 46 Sawkins Road, Rondebosch, 7700 Cape Town, South Africa; Department of Psychiatry, College of Health Sciences, School of Medicine, Addis Ababa University, Addis Ababa, Ethiopia; King’s College London, Institute of Psychiatry, Centre for Global Mental Health, London, UK; Perinatal Mental Health Project, Alan J Flisher Centre for Public Mental Health, Department of Psychiatry and Mental Health, University of Cape Town, Cape Town, South Africa; University of KwaZulu-Natal, Durban, South Africa; Transcultural Psychosocial Organization (TPO) Nepal, Baluwatar, Kathmandu, Nepal; Butabika National Mental Hospital, Kampala, Uganda; Aklilu Lemma Institute of Pathobiology, Addis Ababa University, Addis Ababa, Ethiopia; London School of Hygiene and Tropical Medicine, London, UK; Public Health Foundation of India, New Delhi, India; Sangath, Goa, India; Alan J Centre for Public Mental Health, Department of Psychology, Stellenbosch University, Stellenbosch, South Africa

**Keywords:** Maternal mental health, Global Mental Health, Mental disorders, Depression, Puerperal psychosis, Community mental health services, Primary health care, Developing countries, Sub-Saharan Africa, South Asia

## Abstract

**Background:**

The integration of maternal mental health into primary health care has been advocated to reduce the mental health treatment gap in low- and middle-income countries (LMICs). This study reports findings of a cross-country situation analysis on maternal mental health and services available in five LMICs, to inform the development of integrated maternal mental health services integrated into primary health care.

**Methods:**

The situation analysis was conducted in five districts in Ethiopia, India, Nepal, South Africa and Uganda, as part of the Programme for Improving Mental Health Care (PRIME). The analysis reports secondary data on the prevalence and impact of priority maternal mental disorders (perinatal depression, alcohol use disorders during pregnancy and puerperal psychosis), existing policies, plans and services for maternal mental health, and other relevant contextual factors, such as explanatory models for mental illness.

**Results:**

Limited data were available at the district level, although generalizable data from other sites was identified in most cases. Community and facility-based prevalences ranged widely across PRIME countries for perinatal depression (3–50 %) and alcohol consumption during pregnancy (5–51 %). Maternal mental health was included in mental health policies in South Africa, India and Ethiopia, and a mental health care plan was in the process of being implemented in South Africa. No district reported dedicated maternal mental health services, but referrals to specialised care in psychiatric units or general hospitals were possible. No information was available on coverage for maternal mental health care. Challenges to the provision of maternal mental health care included; limited evidence on feasible detection and treatment strategies for maternal mental disorders, lack of mental health specialists in the public health sector, lack of prescribing guidelines for pregnant and breastfeeding women, and stigmatising attitudes among primary health care staff and the community.

**Conclusions:**

It is difficult to anticipate demand for mental health care at district level in the five countries, given the lack of evidence on the prevalence and treatment coverage of women with maternal mental disorders. Limited evidence on effective psychosocial interventions was also noted, and must be addressed for mental health programmes, such as PRIME, to implement feasible and effective services.

## Background

Maternal and child health are global priority areas for public health interventions. However, despite explicit inclusion in the Millennium Development Goals, the target reductions of maternal and child mortality have not been achieved in many low- and middle-income countries (LMICs) [1]. Accumulating evidence suggests that the neglected issue of maternal mental health may be contributing to this failure [2, 3]. Indeed, when indicators of morbidity and disability are taken into account, mental and behavioural disorders are the largest contributors to disease burden in women of childbearing age [4].

A recent systematic review reported that approximately 16 % of women experienced antenatal depression and 20 % postnatal depression in LMICs - with substantial variations across settings [5]. For example, the reported prevalence of postnatal depressive symptoms in rural Ethiopia was as low as 5 % [6], while in a peri-urban informal settlement in South Africa, a prevalence of 35 % of postnatal depression was found [7]. Risk factors for perinatal depression are well documented [5], and include poverty, lack of emotional and practical support, intimate partner abuse and HIV status [5, 8, 9].

Untreated antenatal depression is also a strong predictor of postnatal depression [10]. Moreover, a mother can be at risk of suicide if her mental illness, especially puerperal psychosis and severe depression, is left untreated [11, 12]. Other consequence of untreated antenatal depression include effects on obstetric outcomes and birth complications [13, 14], on foetal development and neonatal outcomes [15–17], child malnutrition [18], as well as on the child’s behavioural [19, 20], emotional and cognitive development [20, 21].

A core strategy to improve maternal mental health in LMICs, and ultimately to improve maternal and child health, is the integration of mental health into maternal and child health programmes [3]. Task-sharing and stepped-care approaches have been advocated as strategies to facilitate the integration of mental health care into such programmes [3, 22, 23]. There is preliminary evidence which indicates that these are effective in improving outcomes for women with maternal mental disorders and their children in LMICs [24, 25]. These strategies are also employed in the mhGAP Intervention Guide, developed by the World Health Organization (WHO), which provides guidelines on the provision of evidence-based treatments by non-specialist primary health care (PHC) providers in LMICs [26].

The Programme for Improving Mental Health Care (PRIME) is a research consortium, initiated in response to the 75 % mental health treatment gap reported in LMICs [27], which aims to generate evidence on the implementation and scaling up of treatment programmes for priority mental disorders, including maternal mental disorders, in primary and maternal health care contexts in districts in Ethiopia, India, Nepal, South Africa and Uganda [28]. One of PRIME’s objectives is to improve coverage of treatment of mental disorders by adapting, implementing and evaluating WHO’s mhGAP intervention guide. During the inception phase of PRIME, a situation analysis was conducted in each of the five PRIME study sites, to assess existing mental health services for the general population and identify country-specific and cross-country factors relevant to the development and implementation of integrated district-level mental health care [29].

The overall aim of this study was to report the findings of a second situation analysis in the PRIME study sites on maternal mental health. The situation analysis aimed to inform the development of an integrated maternal mental health service. Specifically, we aimed to (i) report on the prevalence of priority maternal mental disorders, (ii) describe the extent to which policy, plans and services for maternal mental health exist, and (iii) describe contextual factors that could influence maternal mental health and the delivery of maternal mental health care within primary care settings. The situation analysis focused on the PRIME priority maternal mental disorders: maternal depression (perinatal depression and depression in mothers of young children), puerperal psychosis and alcohol use disorders (AUDs) during pregnancy.

## Methods

### Research design and setting

This cross-sectional situation analysis of maternal mental health reports secondary data from the districts of Sodo (Ethiopia), Chitwan (Nepal), Sehore (India), Dr Kenneth Kaunda (Dr KK; South Africa) and Kamuli (Uganda). The situation analysis relied mostly on information available in the public domain. Sources included health surveillance data, research publications and personal communication with PRIME investigators, who are experts in the field of public health and maternal mental health.

The PRIME districts offered different opportunities for the development and implementation of the district plans to integrate mental health into maternal health care, and presented a wide range of geographical, demographic, social and cultural profiles. A comprehensive overview of each district is provided elsewhere [29]. Briefly, district populations differed widely, from 162,000 in Sodo (Ethiopia) to 1,300,000 in Sehore (India). Most of the districts’ population lived in rural areas, besides Dr KK (South Africa), where approximately 85 % lived in highly dense urban settings. Each district was characterised by a diversity of ethnicities, religions and languages. Literacy was particularly low in Ethiopia (21.5 %) and in Uganda (62 %), and ranged between 74 and 88 % in India, Nepal and South Africa. Lack of infrastructure was a problem across districts, especially in Sodo (Ethiopia), with poor access to clean water, sanitation or electricity, though Dr KK (South Africa) was well resourced in comparison to the other districts.

### Data collection

The situation analysis focused on the following four domains:

1. Maternal mental health (prevalence of priority maternal mental disorders and impact on maternal and child health)
2. Maternal mental health services (policies and plans, availability of specialised maternal mental health services and personnel, treatment coverage)
3. Maternal health services (coverage, location and staff involved in antenatal, delivery and postnatal care); and
4. Relevant context (task-sharing opportunities; explanatory models of maternal mental disorders, and cultural practices relating to pregnancy and child birth; health-related factors, such as intimate partner violence, HIV, illicit substance use during pregnancy; availability of screening tools, local evidence for effective psychosocial interventions)

Data were collected in three phases. The first consisted of extracting information relevant to maternal mental health, from the data collected between October and December 2011, using the PRIME situation analysis tool. This tool was developed by the PRIME consortium, to collect secondary data, needed for the planning of integrated mental healthcare in the PRIME districts (http://www.prime.uct.ac.za/images/prime/PRIME_Final_Situational_analysis_Tool.pdf). The tool focused on factors required for the implementation of WHO’s mhGAP intervention guide [30], with some items taken from the WHO Assessment Instrument for Mental Health Systems (WHO-AIMS) [31]. It comprised six sections: context, mental health policies and plans, mental health treatment coverage, district level health services, community, and monitoring and evaluation. More information on the development of the tool is provided elsewhere [29]. The situation analysis tool was completed by project coordinators and research staff from the PRIME countries.

Data were then collated into four tables, one for each domain. The second phase, conducted from July to September 2014, consisted of asking PRIME research coordinators and officers to update any outdated data. The third and final phase, between October 2014 and February 2015, consisted of complementing any missing information and assessing data accuracy and quality. The evaluation of the trustworthiness of the findings from published paper was carried out using critical appraisal principles. For unpublished data, an attempt was made to ensure correctness by triangulating sources of data and going back to individuals within the PRIME research team with expert knowledge.

A pragmatic approach was taken when reporting the data: where available, data from the districts were reported. Alternatively, information from neighbouring districts, regional or national data were provided where the data were thought to be generalizable to the PRIME district. Data from these sources were clearly differentiated when reporting the results.

### Ethical considerations

Only data available in the public domain were reported in the situation analysis, and PRIME investigators were consulted within their professional capacity. For this reason, ethical approval was not required for this study. Ethics approval was obtained for the overall PRIME study from the Human Research Ethics Committee at the Faculty of Health Sciences, University of Cape Town (HREC Ref 412/2011).

## Results

### Maternal mental health

Prevalence data were not available at district level in the PRIME countries (Table 1). Prevalence estimates were available for neighbouring districts, however, such as Butajira in Ethiopia, and Latipur in Nepal, or for other regions of the PRIME countries. Prevalence estimates for antenatal and postnatal depression varied considerably. Community studies reported a relatively low postnatal prevalence in Butajira district (Ethiopia; 4.6 %) [6] and in rural Nepal (9.8 %) [32], using the Self Report Questionnaire (SRQ) [33] and the General Health Questionnaire [34], respectively. This is in contrast with the prevalence of 31 % reported in a peri-urban settlement in the Western Cape (South Africa) [7, 35], using a structured clinical interview (SCID; [36]). Facility-based prevalence of postnatal depression, on the other hand, was relatively low in Uganda (6.1 %) [37], and ranged from 3.1 to 19.4 % in Nepal [38–40], using the Edinburgh Postnatal Depression Scale [41]. With the same screening tool, a prevalence of 35.5 % was reported in India [42]. The prevalence of depression was usually higher during pregnancy. Facility-based studies in a rural sub-district of KwaZulu Natal (South Africa) and in Northern Uganda reported an antenatal prevalence of 35.8 % [43] using a structured clinical interview, and 49 % [44] using the Epidemiological Studies Depression Scale [45], respectively. In Butajira district (Ethiopia), symptoms of antenatal common mental disorders were present in 12 % of pregnant women in the community, using the SRQ [14].

**Table 1:** Maternal mental health across the PRIME districts

	Ethiopia	India	Nepal	South Africa	Uganda
Prevalence of antenatal depression	12.0 % [14]^a^	9.2 % [46]^b^; 16.2 % [47]^b^	Unknown	49 % [43]^b^	35.8 % [44]^b^
Prevalence of postnatal depression	4.6 % [6]^a^	23 % [48]^b^; 19.8 % [47]^b^ 24.5–35.5 % [42]^b^	3.1–4.9 % [38]^b^ 9.8–19.4 % [32, 39, 40]^b^	35 % [7]^b^ 31.7 % [35]^b^	6.1 % [37]^b^
Continuity of antenatal depression onto postnatal depression	21.4 % [6]^a^	65.5 % [47]^b;^ 42 % [48]^b^	19 % [50]^b^	23.2 % [49]^b^	Unknown
Prevalence of puerperal psychosis	Unknown	Unknown	Unknown	0.3 % [67]^b^	Unknown
Prevalence of alcohol consumption during pregnancy	5 % (weekly use) [14]^a^	5.8 % (among general women population) [65]^b^	9.4 % [60]^b^	34 % (urban) and 46–51 % (rural) [62]^b^; 19.9 % [63] ^b^	25 % [64]^b^
			15 % [61]^b^		
Evidence of impact of perinatal depression on mother	Disability [55]^a^, prolonged labour [14]^a^	Disability and increased health service use [48]^b^	Unknown	Unknown	Unknown
Evidence of impact of perinatal depression on child	Increased diarrhoeal episodes [56]^a^ and mortality [51] ^a^; impaired development [52]^b^; no association with malnutrition [58]^c^ [57]^a^	Malnutrition [20, 53]^b^; impaired cognitive development [20]^b^	Unknown	Poor attachment [54]^b^ and mother-child relationship [7]^b^; no association with malnutrition [59]^b^	Unknown
Prevalence of foetal alcohol spectrum disorders	Unknown	Unknown	Unknown	59.3–91.0 per 1000 births [66]^b^	61 per 1000 birth defects [127]^c^
Evidence of impact of puerperal psychosis on the mother and child	No studies	Suicidal ideation (38 %) and attempted 0suicide (15 %); ideas of harm to infant [68]^b^	Unknown	Unknown	Unknown

^a^Data from a different district, but same region as PRIME district; ^b^Data from a different region in the PRIME country; ^c^National data

The prevalence of antenatal depression reported in India ranged between 9.2 % [46] and 16.2 % [47], which was lower than in other countries, but continuity of depression from antenatal to postnatal period was notable: Chandran and colleagues [47] reported that 65.5 % of women with antenatal depression continued to suffer from depression at 6–12 weeks postpartum in the area of Tamil Nadu. This was the case for 42 % of women in Goa [48]. In Butajira (Ethiopia), antenatal symptoms of depression persisted after birth for 24 out of 112 women (21 %), and 46 % (20 out 44 women) of postnatal depression had onset after birth [6]. A large cohort study in Johannesburg, South Africa, also found that 23.2 % of women with depressive symptoms at six months postpartum had prior symptoms during pregnancy [49]. Finally, in a mixed facility and community study in Latipur district (Nepal), 19 % of women who reported being depressed during pregnancy screened positive for psychological distress at 6 weeks postpartum [50].

The adverse effects of perinatal depression on mother and child outcomes, commonly indicated in the literature, were also reported in the PRIME countries [7, 14, 20, 48, 51–56]. However, the association between perinatal depression and child malnutrition was not found in Ethiopia [57, 58] or South Africa [59].

Data on alcohol use disorders in pregnant women were also limited. Evidence was available for pregnant women’s alcohol consumption in Ethiopia, Nepal, South Africa and Uganda with wide variations in prevalence [14, 60–64]. A study conducted in the Western Cape in South Africa reported alcohol consumption by a third of pregnant women in the urban area, and by approximately half of pregnant women in rural areas [62]. A cross-sectional study of urban women in Kampala (Uganda), found that 25 % of women still consumed alcohol, at least monthly, after finding out about their pregnancy [64]. Alcohol consumption was lower in Ethiopia and Nepal. In Butajira (Ethiopia), 5 % of pregnant women drank alcohol weekly [14]. Though no evidence was available for alcohol consumption in pregnant women in India, a 2005 WHO report reported that 5.8 % of women consumed alcohol in general [65].

Little evidence was available on the impact of alcohol use disorders on child health outcomes in the specific PRIME countries. However, a high prevalence of foetal alcohol syndrome was reported in the Western Cape, South Africa [66]. This region is known to have a particularly high prevalence, and may not be representative of Dr KK (North West Province).

Information was not available on the prevalence or health impact of puerperal psychosis in the five PRIME countries. One study conducted in Johannesburg in South Africa reported that 0.3 % of women attending an antenatal and HIV clinic, and who were initiated on antiretroviral treatment, were later diagnosed with postpartum psychosis [67]. Another study reported suicidal ideation and attempted suicide in women diagnosed with puerperal psychosis in Bangalore (India), which were associated with ideas of harm to the infant [68].

### Maternal mental health services

Mental health policies and strategic plans, which include maternal mental health, were in place in India and South Africa (Table 2). The new Mental Health Policy Framework and Action Plan for South Africa (2013–2020) [69], which had not yet been implemented, included treatment programmes for maternal mental health as part of the routine antenatal and postnatal care package, programmes to reduce alcohol and substance misuse during pregnancy and programmes in infancy and early childhood to increase maternal sensitivity and infant-mother attachment. The Action Plan also specifically stipulated routine indicated assessment and management of common mental disorders in priority PHC programmes such as antenatal care, postnatal care, and family planning.

**Table 2:** Maternal mental health services across PRIME districts

	Ethiopia	India	Nepal	South Africa	Uganda
Organisational context for maternal mental health (MMH) care					
National mental health policy which includes MMH	Yes	Yes	No	Yes	Yes
National mental health plan which includes MMH	Plan under development, and will include maternal mental health	No mental health plan	No mental health plan	Yes [69]	No mental health plan
Maternal mental health services					
Dedicated MMH service	No	No	No	No	No
Existing MMH care	No, referral to outpatient psychiatric unit (30 km away) or inpatient care (100 km away)	No, referral to mental health professionals posted at district mental health program or at tertiary care centre	No, mental health services available in the district hospital from the general psychiatrist unit	No, referral to PHC psychologists and psychiatrist; mental health services available at district hospitals	No, referral to regional hospital (64 km away)
Prescribing guidelines for psychotropic medication for pregnant & breastfeeding women	No	No	No	No	No
Proportion of perinatal women with disorder in contact with services					
Antenatal depression	Unknown (low)	Unknown	Unknown (low)	Unknown	Unknown
Postnatal depression	Unknown (low)	Unknown	Unknown (low)	Unknown	Unknown
Puerperal psychosis	Unknown	Unknown	Unknown	Unknown	Unknown

Despite the existence of national mental health policies in Ethiopia and Uganda, which considered pregnant women as a vulnerable group, neither policies had plans which included maternal mental health as warranting special attention or focus. India’s mental health policy did not directly address maternal mental health, but did mention the importance of a healthy mother-child bond.

None of the countries reported a dedicated maternal mental health service, and the availability of mental health specialists was either limited, or non-existent in PHC facilities. Instead, perinatal women were required to access mental health care from specialist services, including psychiatric units in general hospitals, either within the district (Chitwan and Dr KK), or from neighbouring districts (Sodo and Kamuli). In-patient psychiatric care was also available in Addis Ababa, over 100 km away from Sodo (Ethiopia). In India, women were referred to mental health professionals posted at district mental health programmes or at tertiary care centres. No information on the treatment coverage of perinatal women with mental disorders was available, however.

Besides South Africa, where 20 % of nurses’ training focuses on mental health [70], pre-service and in-service training of maternal healthcare staff in mental health was limited (more information on the extent of the PHC staff training in mental health is available in Hanlon et al.’s situation analysis [29]). Limited human resources were therefore available to provide mental health care at PHC facilities.

### Maternal health services

Antenatal and postnatal care was delivered as part of PHC for all PRIME countries (Table 3). However, maternity clinics were likely to be separate from the general health care clinics in Sodo (Ethiopia) and Kamuli (Uganda). Antenatal and postnatal care were typically provided by doctors, health officers, nurses or midwives in primary health centres, across the PRIME districts. Community health workers also performed home-based check-ups after birth in Sodo (Ethiopia) and during and after pregnancy in Sehore (India) and Chitwan (Nepal). South Africa’s recent new PHC care plan included home-based postnatal visits via outreach teams [71], however implementation was still inconsistent. The place of delivery was also diverse across the PRIME districts, and tended to be home-based in Ethiopia, India and Nepal, but facility-based in South Africa and Uganda.

**Table 3:** Maternal health services across PRIME districts

	Ethiopia	India	Nepal	South Africa	Uganda
Level of integration of maternal health services with general health services	ANC & PNC integrated in PHC; usually a separate antenatal clinic, run by midwife when present, otherwise by nursing staff	Integrated, provided at both primary and community health centres; facility within centres for examination and safe delivery	Antenatal care, delivery and postnatal services are major services provided within the PHC centres and health posts	ANC & PNC at all clinics with referral systems to general health care; deliveries at district level & some community health centres	Maternity units, run by midwives, are within primary health centre compound, but separate from general health clinics
Attendance for at least one antenatal care (ANC) visit	42.5 % [72]^c^	79.5 % [128]^d^	84.8 % [75]^c^	95.9 % [73]^d^	92.7 % [129]^d^
At least four ANC visits	19.1 % [72]^c^	40.7 % have at least 3 ANC visits [128]^d^	50.1 % [75]^c^	Average 3.5 visits [73]^d^	47.9 % [129]^d^
Average gestation at first ANC visit	5.2 months [72]^c^	3.8 months [128]^c^	3.7 months [75]^c^	40.2 % attend before 20 weeks gestation [130]^c^	5.1 months [131]^c^
Delivery in health facility	5.6 % [72]^d^	26.2 % [128]^d^	35.3 % [75]^c^	84.6 % [73]^c^	86.8 % [129]; 34 % [132]^a^
Attendance of postnatal care (PNC)	5.5 % within 2 days [72]^d^	28.5 % within 2 days [128]^d^	44.5 % within 2 days [75]^c^	52.5 % within 6 days [73]^c^	35.5 % within 2 days [131]^d^
Location of postnatal care	Health centres	Hospitals, primary and community health centres; also provided during home visits	District hospital, primary health clinics, health posts and sub-health posts; home visits also performed	Mostly health-facilities; Limited home visits by CHW for immunization, nutrition and general health information	Health facility
Staff involved in delivery of antenatal care	Health centre: midwives, health officers and trained nurses; Health posts: health extension workers (HEWs)	Health facility: medical officer, auxiliary nurse midwives (ANM) & ASHA^b^; Community: community health workers (CHW)	Health facilities: doctors, nurses and ANM; Community: female community health volunteers (FCHV)	Doctor (at least one visit), nurses and midwives	Doctors, midwives, nursing assistants
Staff involved in delivery of postnatal care	Health centre: nurses; Health posts and community: HEWs	Health facility: medical officer, auxiliary nurse midwives and ASHA^b^; Community: CHW	Health facilities: doctors, nurses and ANM; Community: FCHV	Nurses	Health facilities: doctors, nurses and midwife; community: traditional birth attendant [131]^d^

^a^Data from a different district, but same region as PRIME district; ^b^ Accredited Social Health Activists: community health workers instituted by the government of India’s Ministry of Health and Family Welfare (MoHFW) as part of the National Rural Health Mission; ^c^National data; ^d^Regional data

Attendance for basic antenatal care varied considerably but was relatively high across countries, ranging between 42.5 % in Ethiopia [72] to 96 % in South Africa [73]. Attendance of at least four antenatal care visits, a WHO recommendation [74], was particularly low in Ethiopia, which may be due to the majority of women in Ethiopia delivering at home (94.5 % in the SNNP region). Regional and state data suggested that attendance of postnatal care was generally lower than that of antenatal care across countries, reaching approximately 53 % [73] and 45 % nationally in South Africa and Nepal, respectively [75]. Rates were lowest (5.5 %) in the SNNP region (Ethiopia) [72].

### Relevant context

Staff at PHC clinics reported being in favour of delivering maternal mental health care in Ethiopia [76] and South Africa [77], but lacked confidence in managing mental health problems, even when they received mental health training [78, 79] (Table 4). Stigmatising attitudes among staff towards individuals with mental illness were also reported, especially in Uganda and South Africa [77, 80, 81].

**Table 4:** Relevant context

	Ethiopia	India	Nepal	South Africa	Uganda
Availability of health sector personnel to deliver psychosocial interventions	No	No	No	No	A few (insufficient) psychiatric nurses available in health centres
PHC staff attitudes towards delivering mental health care	General positive attitude; interest in delivering mental health care, greater with higher level of general health training. Need for more training; Poor knowledge of causes of mental disorders [76]^a^	Lack of training on mental health and awareness [133]	Lack of training on mental health and awareness [134]^c^	Nurses think they offer good service to mentally ill patients; doctors feel psychiatric nurses are essential for good mental health care; negative attitudes and stigma towards patients with mental illness, and managers are not supportive [77]^d^ [80]^b^	General negative attitude; health workers stigmatize patients with mental disorders and health workers taking care of them [81]^b^
Community explanatory models of maternal mental disorders	Common perinatal mental disorders attributed to poverty, marital problems or other interpersonal difficulties [82]^a^; psychosis and severe mental illness attributed to supernatural causes [86]^a^	Depression attributed to economic and marital difficulties [83, 84]^a^	Distress perceived as ‘tension’, linked to limited autonomy and perceived duty towards family; women with symptoms of distress also sometimes labelled as ‘witch’ [32]^b^	Poverty, food and financial insecurity, partner rejection, infidelity and general lack of support; medical intervention is not considered appropriate [85]^b^	Attributed to witchcraft & spirits; psychosis can only be treated by traditional African healers [87]^b^; in Ganda culture, puerperal psychosis blamed on supernatural spirits due to promiscuity of the woman during pregnancy [88]^b^
Known cultural practices for pregnant and postnatal women	Cultural taboos on leaving the home following childbirth – confinement for 40 days (for girls) and 80 days (for boys). Health care access dependent on husband’s approval and financial support [82]^a^	Pressure to remain in house during pregnancy and during post-natal period; access to health care services dependent on will of husband and mother in law; dietary restrictions during pregnancy and postnatal period [90]^b^	Childbirth seen as natural event needing no medical assistance; in remote areas pregnancy associated with “shame” and not readily exposed in front of others [135]^b^; role of husband to make financial and transport arrangements for delivery [89]^b^	In some rural areas, confinement (4–6 weeks), not allowed to participate in household activities and duties, sometimes sent home to be under the care of own mothers and family members. Confinement also useful to protect the mother from evil spirits [91]^b^	Own mother or mother-in-law moves in to support the new mother during the first month after giving birth
Illicit substance use during pregnancy	Khat: 12.9 % (weekly) [14]^a^	Unknown	Unknown	Methamphetamine: 8.1 % [63]^b^	Unknown
Prevalence of HIV among pregnant women	6.4 % (urban) [137]^e^; 9–12 % (urban) and <2 % (rural) [94]^a^	0.32 % [96]^d^	0.07 % [95]^c^	36.0 % [137]	6.5 % [138]^c^
Prevalence of intimate partner violence against women	Lifetime: 71 %; past year: 54 % [92]^a^	Lifetime: 38 %; past year: 42.5 % [139]^d^	Lifetime: 33.6 %; past year: 17 % [75]^c^	Past year: 31 % [140]^c^	Past year: 29 % [141]^b^
Validated screening tools for antenatal depression	Yes, but positive predictive value very low^e^	EPDS & K10 in rural setting [104]^a^	No	K10/K6 [103]^b^;EPDS [142]^b^ 3-item tool [105]^b^	CES-D [44]^b^
Validated screening tools for postnatal depression	SRQ in rural setting [33]^a^; K10/K6 and EPDS in urban setting [100]^b^	SRQ [102]^a^ EPDS and BDI [42] ^a^	EPDS [39]^a^ SRQ [102]^a^	EPDS [101]^b^ SRQ [102]^b^	None
Validated screening tool for AUDs	No	AUDIT [97]^a^	AUDIT [98]^a^	AUDIT [99]^b^	AUDIT-C [143]^b^
Screening tools used in clinical practice for perinatal depression or AUD	No	No	No	No	No
Evidence for a culturally relevant psychosocial intervention for depression in perinatal women	None	Maternal self-help groups [108]^a^	None	Counselling as part of stepped care [23]^b^; parenting support and guidance [107]^b^	None
Evidence for culturally relevant psychosocial intervention for AUD	None	None	None	None	None

^a^Data from a different district, but same region as PRIME district; ^b^Data from a different region in the PRIME country; ^c^National data; ^d^Regional data; ^e^Personal communication with F Girma (2014)

Explanatory models of mental illness across PRIME countries often attributed common perinatal mental disorders to social and economic adversity, such as poverty, marital and interpersonal difficulties [32, 82–85]. In Nepal, women with symptoms of distress were also sometimes labelled as a ‘witch’ [32]. Psychosis, on the other hand, was attributed to supernatural causes, witchcraft or ancestors in Ethiopia [86] and Uganda [87, 88]. Rarely did explanatory models include biomedical concepts in Ethiopia [86]. These led to stigmatising attitudes towards perinatal women with mental disorders.

Cultural practices in Ethiopia, such as confinement of the mother and infant for 40–80 days after birth [82], as well as the need for the husband’s approval and financial support for any health care access in Ethiopia, India and Nepal [82, 89, 90], may also prevent women from accessing mental health care. Confinement for four to six weeks was also a common practice in rural areas in South Africa to protect the mother from evil spirits [91]. In Nepal, giving birth was seen as a natural event requiring no medical assistance, which corroborates the lower rate of delivery in health facility reported nationally [75].

Health-related factors which often contribute to maternal mental disorders were also common in the PRIME countries, according to national health surveys (Table 4). Intimate partner violence (IPV) was highly prevalent across all PRIME countries: the highest lifetime prevalence was reported in Butajira, Ethiopia (71 %) [92]. District data on HIV prevalence were available for Dr KK (South Africa), which indicated the highest HIV prevalence of 36.0 % across the PRIME countries [93]. Regional or national HIV prevalence in the other four countries varied widely. In the Southern Nations, Nationalities, and People’s Region (SNNPR; Ethiopia), which includes Sodo district, prevalence ranged between less than 2 % in rural areas, to 9-12 % in urban areas [94]. A similar prevalence of 6.5 % was found in Uganda, whereas prevalence was well below 1 % for both Nepal [95] and India [96].

Illicit substance use during pregnancy was also common in Butajira district (Ethiopia) and the Western Cape Province (South Africa). Weekly or daily use of khat was reported in 12.9 % of pregnant women in Butajira district [14]. A recent large cross-sectional study, conducted in 11 public obstetric facilities in greater Cape Town, revealed that 8.1 % of pregnant women tested positive for methamphetamine use (tik) based on biological screening [63]. No information on substance use during pregnancy was available for India, Nepal or Uganda.

Screening tools had been validated in the PRIME countries to detect women presenting with symptoms of depression or alcohol misuse during the perinatal period, however none were in clinical practice in the districts. The Alcohol Use Disorder Identification Test (AUDIT) was the only available and validated screening tool for hazardous drinking in India [97], Nepal [98] and South Africa [99]. Several screening tools were validated for perinatal depression in the PRIME countries, however. The Edinburgh Postnatal Depression Scale (EPDS) was the most widely validated tool to screen for postnatal depression [42, 100], and had already been used in Nepal and South Africa in research contexts [39, 101]. However, the EPDS was not found to be sensitive enough in rural settings in Ethiopia [100], where the Self-Rating Questionnaire (SRQ) was reported as more useful [33]. The SRQ was also validated in India, Nepal and South Africa [102]. The Kessler Questionnaire was also validated for use in urban postnatal women in Ethiopia [100] and in pregnant women in South Africa [103] and India [104]. The Centre for Epidemiologic Studies Depression Scale (CES-D) was the only screening tool validated in Uganda, in a population of pregnant women [44].

A shorter, three-item detection tool for antenatal symptoms of common mental disorder was recently validated in a township of the Western Cape (South Africa) [105], but still needed further field-testing in other South African settings. A Community Informant Detection Tool (CIDT) was also recently developed in Nepal for the identification of depression, psychosis, epilepsy and alcohol use disorder at community level [106], but it had not yet been adapted for pregnant and postnatal women.

Data were limited on the effectiveness of psychosocial interventions in the five PRIME countries. There is evidence from studies in South Africa, however, suggesting that counselling and parental support has beneficial effects in reducing symptoms of common perinatal mental disorders [23], and in improving the quality of the mother-child relationships [107]. One study assessing the effectiveness of maternal self-help groups in East India reported positive child and maternal health outcomes, and a reduction in moderate depression at two years postpartum [108]. However, little improvement in depressive symptoms was found at six weeks, one year and three year postpartum. There was no evidence on the effectiveness of psychosocial interventions for alcohol use disorders in any of the PRIME countries. Generally, health sector personnel were not available to provide these psychosocial interventions, besides in Dr KK (South Africa), where psychologists may be available at primary care facilities.

## Discussion

### Main findings

The present situation analysis in the PRIME districts provided some insight into the national or regional prevalence and impact of target maternal mental disorders. It has also contributed to our understanding of the current health and mental health services in place for perinatal women in the districts, as well as the factors that may influence the delivery of maternal mental health care in primary care settings.

#### Maternal mental health

National or regional data available on antenatal and postnatal depression corroborated international evidence: perinatal depression was prevalent but ranged widely across the PRIME countries. It must be noted that different diagnostic or screening tools were used across community and facility-based studies, which may explain the disparities in the reported prevalence estimates. As a result, estimating prevalence of perinatal depression in the PRIME districts is difficult. As the data suggest, it may be that prevalence does indeed vary widely across PRIME countries, and even across regions within countries.

There was an absence of evidence about the prevalence of puerperal psychosis in the districts, or at national level. More information was available on alcohol consumption in the PRIME countries, however. There is mixed evidence regarding the accuracy of self-disclosure of alcohol use during pregnancy [63, 109]. Yet, even self-reported data suggested that a high proportion of women in the Western Cape (South Africa) and in Kampala (Uganda) consumed alcohol during pregnancy [62, 64]. The fact that the rate of Foetal Alcohol Spectrum Disorders in the Western Cape is amongst the highest worldwide reflects the extent of the problem [66]. A high proportion of illicit substance use during pregnancy was also reported in Butajira district (Ethiopia) and the Western Cape (South Africa). It is unknown, however, whether substance and alcohol use problems are as extensive in Sodo and Dr KK districts specifically. Given the implications of substance use on birth weight [110], neonatal development [111] and on the infant’s health and development [112], as well as on the user’s mood, anxiety and sleep patterns [113, 114], more research is needed to assess the level of substance and alcohol consumption among perinatal women in the PRIME districts and the treatment needs of this population.

#### Maternal health and mental health services

The situation analysis highlights how maternal mental health services in the PRIME districts are sorely lacking. None of the PRIME districts had mental health services dedicated for perinatal women, or had strategies in place in primary health facilities to detect maternal mental disorders. However, referral mechanisms to specialist mental health services, including psychiatric units at district hospitals or to inpatient care at tertiary level, were in place across all five PRIME districts. Unfortunately, lack of information systems for the provision of mental disorders in the district meant that it was not possible to report on the number of perinatal women who were receiving mental health care or who were in contact with psychiatric services. Even with mental health policies in place, the limited human resources trained and able to provide mental health care at primary health care facilities, the stigmatising attitudes still reported by staff towards patients with mental illness, and the lack of prescribing guidelines for psychotropic medication in pregnant and breastfeeding women, all suggest that there was little opportunity for women with maternal mental disorders to be detected and receive mental health care at primary level.

Indeed, besides Nepal, all PRIME countries had mental health policies which referred to maternal mental health, and both South Africa and India had plans which specifically included strategies and guidelines for the provision of maternal mental health. These plans were developed recently, however, and remained to be fully implemented. These were promising measures, which should improve detection and coverage of maternal mental disorders, as well as strengthen maternal mental health information systems.

The situation analysis highlights the diverse settings in which antenatal, delivery and postnatal care is delivered across the PRIME districts. If maternal mental health were to be integrated in PHC, detection, referral and treatment processes would likely need to be tailored to maternal services and adapted across the different PRIME districts; in some districts such as Ethiopia, India and Nepal, detection and referral may be more feasibly integrated in community-level maternal care, whereas for South Africa and Uganda, detection and treatment may be more effective and sustainable if it is facility-based. Home-based interventions would also overcome the service access barriers associated with the cultural practices of restricting mothers to the home. Community health workers tend to predominantly engage in nutrition, immunisation and HIV counselling, so this cadre would also have to be trained to provide early detection of maternal mental disorders.

#### Relevant context

Evidence shows a strong association between mental illness and HIV status, especially during pregnancy [5, 9]. HIV status is often detected during pregnancy, as part of the antenatal services, and there is some evidence suggesting that perinatal women with depression are less likely to attend health care and adhere to antiretroviral medication [115]. Several studies have also reported that women are at increased risk of experiencing IPV during pregnancy [76, 116]. Given the high prevalence of IPV reported across PRIME countries, as well as the high HIV incidence among pregnant women in urban Ethiopia and in South Africa, it is worth considering the integration of maternal mental health into maternal and child health platforms [117]. This would also be a useful way to overcome community stigma, highlighted in the situation analysis.

Other contextual factors, reported in this situation analysis, may influence the identification and treatment of maternal mental disorders and the provision of, and access to maternal mental health services. Though several screening tools for perinatal depression and AUD had been validated in the PRIME countries, none were used in PHC practice. This may reflect lack of political will or lack of communication between researchers and health planners and managers. There is a current debate relating to the effectiveness of routine administration of screening tools in primary care settings, and whether this strategy does improve detection and the provision of mental health care. These tools are often developed for research purposes, and may be cumbersome for routine screening in the context of overburdened health care systems and scarce human resources [118, 119]. Routine screening also entails that standard operating procedures, referral mechanisms and treatment services be in place for individuals screening positive, together with the necessary ongoing quality improvement, supervision and mentoring of PHC staff administering the screening tool [119]. These structures were not in place in the PRIME districts, but are essential if detection of common perinatal mental disorders is to be integrated in PHC.

Strong supervision, monitoring and support structures would also be necessary for the delivery of psychosocial interventions to be at all possible at PHC level in scarce resource settings. This is particularly relevant when evidence suggests that PHC staff report not being able to provide maternal mental health care. Although there is growing evidence from other LMICs indicating that psychosocial interventions can be culturally adapted and are effective at addressing common mental disorders [24, 25, 120], the transferability of these findings to rural settings in low-income countries, and feasibility of these interventions to scarce-resource clinical settings, needs to be demonstrated. To promote these structures in the face of a shortage of mental health specialists in primary care settings, evident across the PRIME districts [29], more innovative approaches may be considered, such as peer support interventions. Also, much less evidence is available on task-shared interventions for alcohol use disorders and psychosis, so more research should be conducted to evaluate short, feasible interventions and rehabilitation programmes for perinatal women.

Pharmacological treatments are typically less resource-intensive, and may be more feasibly integrated at PHC level for the treatment of maternal mental disorders. Current mhGAP guidelines indicate that psychotropic medication can only be initiated and monitored by a mental health specialist. The lack of mental health specialists and national guidelines for prescribing psychotropic treatment to pregnant and breastfeeding women may therefore also pose a challenge to the provision of psychotropic medication for women suffering from puerperal psychosis, severe or non-responding depression in PRIME districts. Moreover, there are potential risks involved with the use of psychotropic medication in pregnant and breastfeeding women, which must be considered in relation to the risks of untreated perinatal depression for the foetus and the child [121, 122]. Though some antidepressants, such as tricyclic antidepressants and fluoxetine, have been shown to have less adverse effects on the health and development of the child [123, 124], best practice requires that a pregnant or breastfeeding woman’s decision to initiate psychotropic medication should be made in consultation with a mental health specialist. Finally, the acceptability of psychotropic medication during pregnancy and breastfeeding, both among women and health care staff, was not reported in the situation analysis, but is likely to differ across the PRIME districts. While medication may be more desirable than psychosocial interventions in India and Uganda, in South Africa, evidence suggests that psychosocial interventions or ‘talking therapies’ are more acceptable than medication for the treatment of depression and alcohol use disorders [125, 126]. This corroborate the explanatory models reported in South Africa by Kathree et al. [85].

### Limitations

The information drawn from the situation analysis provides a partial overview of maternal mental health and services across the five districts, with only limited data available for the specific districts. Information was instead gathered from research studies conducted in other regions of the PRIME countries, with smaller, non-representative samples, and little opportunity to generalise to the PRIME districts.

While authors ensured that the situation analysis was as comprehensive as possible, data collected were dependent on documents available in the public domain, and on the knowledge of the authors and consulted PRIME research teams. Also, several maternal health indicators were extracted from different national reports published at different time points in the PRIME countries, some of which reported ten-year old data. Some indicators were therefore more up-to-date than others.

Despite these limitations, the present study combined secondary data from a variety of sources within a framework of information needed to plan mental health services. Moreover, contextual regional and national data remain useful to understand the context for maternal mental health services, and the potential for planned maternal mental health care to be scaled up to other districts or regions within the PRIME countries.

## Conclusions

The situation analysis highlighted the lack of evidence on the socio-culturally relevant psychosocial interventions available in the PRIME countries. The lack of information on the prevalence and coverage of priority maternal mental disorders in the PRIME districts was also a striking finding, making it difficult to estimate the needs of the perinatal population with mental disorders. It is imperative that any mental health programme include information systems to ensure that detection and treatment coverage of maternal mental disorders can be monitored. Being cognisant of factors which influence access to mental health care, such as those highlighted in this situation analysis, should help future programmes like PRIME to develop more effective detection, referral and treatment strategies, and ultimately contribute to reducing the burden of maternal mental disorders on mothers and children in low-resource settings.

## Abbreviations

LMICs: low- and middle-income countries
WHO: World Health Organization
PHC: primary health care
PRIME: Programme for Improving Mental Health Care
IPV: interpersonal violence
AUDIT: Alcohol Use Disorder Identification Test
EPDS: Edinburgh Postnatal Depression Scale
SRQ: Self-Rating Questionnaire
CES-D: Centre for Epidemiologic Studies Depression Scale
